# Potential Relationship between Cerebral Fractional Tissue Oxygen Extraction (FTOE) and the Use of Sedative Agents during the Perioperative Period in Neonates and Infants

**DOI:** 10.3390/children7110209

**Published:** 2020-11-03

**Authors:** Danguolė Č Rugytė, Loreta Strumylaitė

**Affiliations:** 1 Department of Anesthesiology, Lithuanian University of Health Sciences, 44307 Kaunas, Lithuania; 2 Neuroscience Institute, Lithuanian University of Health Sciences, 50161 Kaunas, Lithuania; loreta.strumylaite@lsmuni.lt; 3 Department of Preventive Medicine, Faculty of Public Health, Lithuanian University of Health Sciences, 47181 Kaunas, Lithuania

**Keywords:** hyperoxia, neonate, infant, benzodiazepines, opioids, cerebral near infrared spectroscopy, fractional tissue oxygen extraction

## Abstract

Fractional tissue oxygen extraction (FTOE) by means of cerebral near-infrared spectroscopy (NIRS) provides information about oxygen uptake in the brain. Experimental animal data suggest that sedative agents decrease cerebral oxygen demand. The aim of the present study was to investigate the association between the cerebral FTOE and the use of pre and intraoperative sedative agents in infants aged 1–90 days. Cerebral NIRS was continuously applied during open major non-cardiac surgery in 46 infants. The main outcomes were the mean intraoperative FTOE and the percentage (%) of time of intraoperative hyperoxia_FTOE_ relative to the total duration of anesthesia. Hyperoxia_FTOE_ was defined as FTOE ≤ 0.1. Cumulative doses of sedative agents (benzodiazepines and morphine), given up to 24 h preoperatively, correlated with the mean intraoperative FTOE (Spearman’s rho = −0.298, *p* = 0.0440) and were predictive for the % of time of intraoperative hyperoxia_FTOE_ (β (95% CI) 47.12 (7.32; 86.92)) when adjusted for the patients’ age, type of surgery, preoperative hemoglobin, intraoperative sevoflurane and fentanyl dose, mean intraoperative arterial blood pressure, and end-tidal CO_2_ by multivariate 0.75 quantile regression. There was no association with 0.5 quantile regression. We observed the suggestive positive association of decreased fractional cerebral tissue oxygen extraction and the use of sedative agents in neonates and infants undergoing surgery.

## 1. Introduction

Major surgery in neonates and infants has been related to worse neurodevelopmental outcomes [1]. The definitive causes of this association are not fully determined, but surgical disease, prematurity, and the potential disturbance of normal physiologic parameters are among the leading ones.

Painful stimulation accompanies every perioperative period. Pain itself has been related to poorer long-term outcomes [2]. Benzodiazepines and opioids are most widely used agents for neonatal and infantile sedation and analgesia [3]. However, negative long-term behavioral effects and the growth retardation of certain brain structures have been associated with the use of these agents in very preterm infants [2,4,5]. Therefore, the effects of sedatives on the central nervous system of neonates and infants are important.

Near-infrared spectroscopy (NIRS) was shown to be a convenient method for monitoring cerebral tissue oxygenation during surgery and intensive care [6]. Along with other central nervous system monitoring tools, it may help to differentiate between brain activity, oxygen delivery, or oxygen consumption-associated clinical scenarios in critically ill patients [7]. NIRS-derived fractional tissue oxygen extraction (FTOE) provides additional valuable information on oxygen uptake in the brain. Changes in FTOE values were shown to be associated with such clinical conditions as the development of intraventricular hemorrhage/periventricular leukomalacia [8,9], hypotension [10], and even unfavorable effects of oxygen treatment [11].

Factors such as arterial blood pressure, hemoglobin level, arterial blood oxygen saturation [12,13,14], and even pain [15,16] can affect the cerebral oxygen supply and influence cerebral tissue oxygenation. On the other hand, cerebral metabolic demand and oxygen uptake can be affected by sedative and analgesic agents used during the perioperative period and intensive care. Thus, balance between oxygen supply and oxygen demand can be reflected in the cerebral oxygenation and FTOE values.

A decreased oxygen uptake with the use of benzodiazepines and opioids was determined in experimental animal studies [17,18]; however, this has not been demonstrated in the clinical setting by the means of cerebral near-infrared spectroscopy. Here, we present the results of the prospective observational trial on the intraoperative cerebral NIRS with the focus on NIRS-derived FTOE in infants undergoing major general surgery. We aimed to investigate the association between cerebral FTOE and perioperative clinical variables, including the use of pre- and intraoperatively administered sedative and analgesic agents.

## 2. Materials and Methods

Neonates and infants younger than 3 months old undergoing open non-cardiac surgery for congenital anomalies or diseases were eligible for the study. Patients were excluded if they had clinical conditions that significantly affected their cerebral oxygenation values. These were Hb < 80 g·L^−1^ or hyperbilirubinemia, bronchopulmonary dysplasia, and neurosurgical disease. Patients with malignancy, sepsis, bone or muscle disease, renal or hepatic insufficiency, or a physical status corresponding to the American Society of Anesthesiologists (ASA) classification 5th class were also excluded.

General anesthesia with tracheal intubation using a sevoflurane in air and oxygen mixture (1:1) was used. During anesthesia, cerebral oxygenation (rSO_2_) monitoring (INVOS^®^, SOMANETICS (Medtronic, Minneapolis, MN, USA)) was continuously applied until wound closure. Before the induction of anesthesia, two pediatric cerebral sensors were placed bilateral to the forehead region and rSO_2_ monitoring was started. Data were captured with a sampling interval of 5 s and recorded every 5 min. Simultaneously, the arterial blood pressure (mean), hemoglobin (Hb), oxygen saturation (SpO_2_), end tidal (ET) CO_2_, and ET sevoflurane concentration in percentages (%) were registered (more methodological details can be found elsewhere [14]). The cerebral FTOE was calculated according to the formula: (SpO_2_-rSO_2_)/SpO_2_ [19,20] for each patient at every 5 min interval of each pair of SpO_2_ and rSO_2_. The main outcome measures were mean intraoperative FTOE and the duration of hyperoxia by FTOE (hyperoxia_FTOE_) during surgery relative to the total duration of anesthesia (% of the time of intraoperative hyperoxia_FTOE_).

Hyperoxia_FTOE_ was defined as FTOE ≤ 0.1, normoxia_FTOE_ as FTOE > 0.1 and ≤0.4, and hypoxia_FTOE_ as FTOE > 0.4 [11,19].

Individual cumulative doses in mg·kg^−1^ were calculated separately for each intravenously (iv) administered sedative agent (diazepam, midazolam, and morphine (opioid)) up to 24 h before surgery. In patients given a combination of two or three sedative agents, the individual cumulative doses of diazepam, midazolam, and morphine were added (assuming the ratio 1:1:1), resulting in the cumulative dose of all sedative agents in mg·kg^−1^ for each patient. Intravenous fentanyl (opioid), oral or rectal forms of benzodiazepines, or morphine were not used preoperatively.

### Statistical Methods

For each patient, mean values were calculated for intraoperative variables: rSO_2_, FTOE, mean arterial pressure, SpO_2_, ET CO_2_, ET % sevoflurane. Shapiro–Wilk test was used to assess the normality of distribution of continuous data. Parameters distributed abnormally are presented as median (range), normally distributed parameters are presented as mean and standard deviation (SD), and categorical and binomial variables are presented as number of patients (%). The Mann–Whitney U test was used for the comparison of abnormally distributed continuous variables, the unpaired t test for normally distributed continuous variables, and the chi square test for categorical variables.

Spearman’s rank correlation was used to assess the associations of FTOE and the % of time of intraoperative hyperoxia_FTOE_ with the cumulative doses of preoperative sedative agents and the demographic and clinical variables. As the main outcome variable, % of time of intraoperative hyperoxia_FTOE_, was distributed abnormally, a univariate and multivariate 0.5 (median) and 0.75 quantile regression analysis [21] was used to assess the predictive value of cumulative doses of preoperative sedative agents and clinical variables for the % of time of intraoperative hyperoxia_FTOE_, calculating regression coefficients (β) and their 95% confidence intervals (CI); *p* < 0.05 was considered statistically significant for all the statistical tests. All the statistical analyses were performed using the STATA/IC 15 software (Stata Corporation LLC, Lakeway Drive, TX, USA).

The study was approved by the Kaunas Regional Biomedical Research Ethics Committee (protocol no BE-2-43, study registration no. NCT02423369 at Clinicaltrials.gov.), and conducted in accordance with the Helsinki Declaration. Informed written parental consent was obtained before the enrollment of each patient.

## 3. Results

The cerebral oxygen saturations were measured in 46 patients. The demographic and clinical characteristics of the studied patients are shown in Table 1. Hyperoxia_FTOE_ during surgery occurred in 69.6% (*n* = 32) of patients, with a duration of 14.7 (0–100)% (median (range)) of time. Hypoxia_FTOE_ was observed in 6.5% (*n* = 3) patients, with a duration of 59 (7.7–65)% of time.

The pattern of administration and the cumulative doses of preoperative sedative agents are shown in Table 2. In all patients, diazepam was administered within 24 h before surgery. Morphine was given by continuous infusion for pain or sedation for no less than 10 h before surgery or as a single dose at a 1 (1–3) hour (median (range)) before surgery to facilitate the induction of anesthesia. Midazolam was given within 3 h before surgery alone or as an adjunct to morphine and diazepam to facilitate the induction of anesthesia.

**Figure 1:**
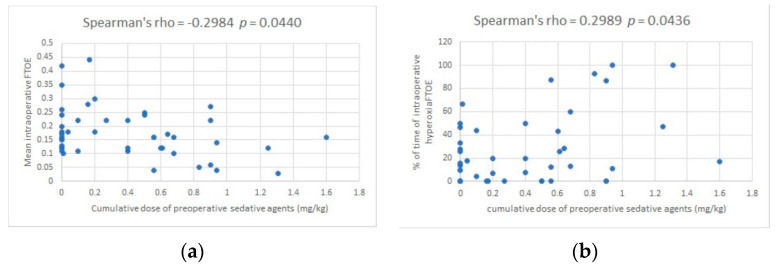
Correlation between the cumulative doses of preoperative sedative agents and cerebral fractional tissue oxygen extraction (FTOE) (by means of near-infrared spectroscopy) (**a**) Correlation between the cumulative doses of preoperative sedative agents and the mean intraoperative FTOE; (**b**) correlation between the cumulative doses of preoperative sedative agents and the % of time of intraoperative hyperoxia_FTOE_ (when FTOE ≤ 0.1).

In order to identify potential associations with intraoperative hyperoxia, we correlated the cumulated doses of sedative agents and demographic and clinical variables with the mean intraoperative FTOE values and the % of time of intraoperative hyperoxia_FTOE_. The cumulative doses of sedative agents moderately correlated with both FTOE (Figure 1a) and the % of time of intraoperative hyperoxia_FTOE_ (Figure 1b), but not with the mean intraoperative rSO_2_ values (Spearman’s rho = 0.258, *p* = 0.084). Patient’s age correlated with the % of time of hyperoxia_FTOE_ (Spearman’s rho = −0.34, *p* = 0.020), but not with FTOE. There were no other significant correlations.

To estimate the potential predictive value, the cumulative doses of preoperative sedative agents and clinical parameters were included into a univariate 0.5 and 0.75 quantile regression model. Only the cumulative doses of preoperative sedative agents were positively predictive for the % of time of intraoperative hyperoxia_FTOE_ by 0.75 quantile regression (Table 3) but not by 0.5 quantile regression.

In order to find out possible confounders associated with the administration of sedative agents preoperatively, we compared the pre- and intraoperative variables between patients given and not given sedative agents. The patients given sedative agents were younger, had undergone predominantly abdominal surgery, had higher preoperative Hb levels, and received less sevoflurane during surgery (Table 4). Therefore, age, type of surgery, preoperative Hb, and intraoperative ET sevoflurane concentration were included into multivariate quantile regression models, along with the cumulative doses of preoperative sedative agents.

During surgery, several other important factors may influence cerebral oxygenation and consequently FTOE. These are arterial blood pressure, intraoperatively administered fentanyl, and ET CO_2_. Although these factors did not differ between the patients given and not given sedative agents preoperatively, it is clinically relevant to include them as possible risk factors into the multivariate regression model. The model including all possible confounding variables revealed that the cumulative dose of preoperative sedative agents significantly predicted the % of time of intraoperative hyperoxia_FTOE_ at the 75th percentile but not at the 50th percentile (Table 5). The above-mentioned multivariate quantile regression models, including possible confounding variables, are shown in Tables S1 and S2. According to our data, the 75th percentile corresponds to a duration of intraoperative hyperoxia_FTOE_ no less than 43.8%, whereas the 50th percentile (median) corresponds to a duration of 14.7%. Thus, it means that cumulative doses of preoperatively administered sedative agents are potentially predictive for a prolonged (43.8% and over) but not short duration of intraoperative hyperoxia.

The in-hospital outcomes did not differ between patients with and without intraoperative hyperoxia_FTOE_ (*p* = 0.264). One of the three patients with intraoperative hypoxia_FTOE_ had bilateral subependymal hemorrhages during the in-hospital follow-up.

## 4. Discussion

The experimental animal data indicate that benzodiazepines and opioids reduce cerebral oxygen uptake [17,18]. Here, we demonstrate a suggestive positive association between the use of sedative agents and decreased cerebral oxygen extraction by means of NIRS.

Recently, Weber and Scoones described increased cerebral NIRS values in ventilated neonates who were given midazolam for sedation [6], however no further details were provided. We included the doses of sedative agents administered up to 24 h before surgery. Assuming a prolonged elimination half time of these agents in neonates [22,23,24], their effects were highly pharmacologically reliable intraoperatively. Prolonged sedative effects of benzodiazepines and morphine were demonstrated in studies recording electroencephalography (EEG) in preterm as well as surgical infants [25,26,27]. The results of our study and study by Weber and Scoones can be explained by the depressant effects of benzodiazepines and opioids on electrocortical activity, as shown by means of EEG. During states of diminished electrocortical activity, a decrease in FTOE and increase in rSO_2_ as markers of reduced oxygen uptake were demonstrated during the simultaneous use of NIRS and EEG for brain function monitoring in term and preterm neonates with different pathological conditions [7], in preterm neonates during the first hours after birth [28], as well as in neonates sedated with propofol [29]. As amplitude-integrated EEG is becoming a bed-side tool during neonatal intensive care, studies appeared showing that the electrocortical activity in preterm neonates may be related to later neurodevelopmental outcomes [30]. Therefore, the effects of sedative agents are important.

Apart from sedative effects, inhalational anesthetic sevoflurane, which was used during the maintenance of anesthesia in our patients, depending on the administered concentration may affect the cerebral blood flow through the direct effect on cerebral blood vessels or through the effect on the systemic arterial blood pressure, thus affecting the cerebral oxygen supply, cerebral oxygenation, and consequently FTOE [31]. The observed decreased doses of sevoflurane in patients given preoperative sedative agents may reflect both the effects of preoperative sedatives as well as a worse patient condition, which might have been associated with younger age and/or worse abdominal pathology. Younger age and abdominal pathology may per se affect cerebral oxygenation due to potential differences in the cerebral blood flow autoregulation, greater metabolic disturbance, and pain, a condition which was also linked with changes in cerebral oxygenation [15,16]. The sedative effects of intraoperatively administered opioid-fentanyl must also not be ignored. Nevertheless, all these possible confounding factors included into a multiple regression model did not change significantly the predictive value of cumulative doses of preoperatively administered sedative agents on the prolonged duration of hyperoxia.

Carbon dioxide is a potent factor of cerebrovascular reactivity and thus oxygen delivery to the brain. Other investigators, though, did not find significant association between partial CO_2_ pressure in blood (p CO_2_) and FTOE [28]. ET CO_2_ during neonatal and infantile surgery is highly unreliable due to considerable dilution of exhaled gas with fresh gas. A more precise method would have been measurement of p CO_2_. Invasive methods, such as the placement of arterial cannulae, allow us to obtain multiple p CO_2_ measurements. However, arterial cannulae were not present in our patients. For these reasons, ET CO_2_ included into the model may carry a certain bias.

Both cerebral hypoxia and hyperoxia have been related to worse conditions in severely ill neonates and infants [7,32]. Despite the association of sedative agents with hyperoxia_FTOE_ in our study, the in-hospital outcomes did not differ between patients with and without hyperoxia_FTOE_. This is in accordance to the observation of SafeBoosC trial, where no major neurological complications nor deaths were associated with cerebral hyperoxia within 72 h after birth [32]. The same trial also did not detect differences in neurodevelopmental outcome at the age of 2 years between former preterm infants treated and not treated for abnormal cerebral oxygenation, although the incidence of hyperoxia was low compared with the incidence of hypoxia [33].

### Limitations

Although directly, related rSO_2_ and FTOE values may provide us with different and additive information. We based our analysis on FTOE, but not rSO_2_ values, as there was no correlation between cumulative doses of sedative agents and the mean intraoperative rSO_2_. The categorization of hyperoxia_FTOE_ selected by us was based on previous publications in preterm infants or neonates in the immediate transition period after birth. Whether these thresholds are applicable to term infants up to 3 months of age is unknown. However, the cumulative doses of sedative agents correlated not only with the duration of time with FTOE ≤ 0.1, but also with mean intraoperative FTOE, showing that higher cumulative doses of sedative agents were positively associated with less cerebral oxygen extraction. We based our analysis on the % of time (or duration) of intraoperative hyperoxia_FTOE_, as this measure more adequately than the mean intraoperative FTOE reflects the intraoperative variability in FTOE values.

The coadministration of benzodiazepines and opioids occurred in almost half of patients given sedative agents. Although these two classes of sedatives have a different pharmacology, it was shown that coadministration results in functional but not pharmacological synergism, which is also not pharmacokinetics-dependent [34]. However, information regarding equipotent doses of different benzodiazepines and opioids is inconsistent, inadequately supported with clinical evidence, and therefore of limited utility [35,36]. Furthermore, although, these two classes of agents reduce oxygen uptake in brain cells, scientific data on the comparative magnitude or clear association with the administered doses are lacking [37]. For all the above-mentioned reasons, we found it appropriate to analyze the effect on cerebral oxygenation calculating the cumulative doses of diazepam, midazolam, and morphine according to the ratio 1:1:1.

This was an observational trial, describing present clinical practice in a patient cohort of limited size and undergoing surgery. Under these circumstances, the effects of sedative agents interplay with many other significant factors. Although we tried to take these factors into account, our results may not be applicable to all neonates and infants. We also had no possibility of measuring the cerebral oxygenation at baseline before any sedatives were administered, therefore the relationship between the intraoperative hyperoxia_FTOE_ and the preoperative dose of sedative agents should be interpreted with caution.

## 5. Conclusions

We observed a suggestive positive association of decreased fractional cerebral tissue oxygen extraction by means of cerebral near-infrared spectroscopy and the use of benzodiazepines and opioids in surgical neonates and infants up until 3 months of age. In order to better understand the magnitude of the effect of sedative agents on infants’ brain tissue oxygenation within the complex clinical scenarios and the potential relationship with outcomes, future clinical trials in larger and more homogenous populations are required. Additionally, using techniques allowing us to directly observe the effects of sedative agents on the electrocortical activity would be helpful in quantifying the effects on the infantile brain.

## Figures and Tables

**Table 1:** Demographic and clinical characteristics of the studied infants (*n* = 46).

Variable	Median (Range) or *n (%)*
Age (days)	10.5 (0–90)
Male sex	27 (58.7)
Weight (g)	3366.33 (878.44) ^1^
Gestation (weeks)	37.67 (2.41) ^1^
Preterm infants	15 (32.61)
ASA class	
1	2 (4.35)
2	21 (45.65)
3	17 (36.96)
4	6 (13.04)
Type of surgery	
Abdominal	24 (52.17)
Other	22 (47.83)
Patients on preoperative mechanical ventilation	6 (13.04)
Duration of surgery (min)	80 (30–260)
Intraoperative rSO_2_ (%)	79.76 (8.89) ^1^
Intraoperative FTOE	0.16 (0.03–0.44)

^1^ Mean (SD).

**Table 2:** The pattern of administration and the cumulative doses of sedative agents given within 24 h before surgery.

Sedative Agent	*n* (%)	Cumulative Dose, mg·kg^−1^, Median (Range)		
		Diazepam	Midazolam	Morphine
Diazepam	9 (19.57)	0.5 (0.1–1.6)		
Midazolam	4 (8.7)		0.12 (0.01–0.4)	
Morphine	3 (6.52)			0.17 (0.1–0.4)
Diazepam + midazolam	2 (4.35)	0.9 (0.9–0.9)	0.04 (0.04–0.04)	
Diazepam + morphine	9 (19.57)	0.44 (0.08–1.3)		0.17 (0.01–0.58)
Midazolam + morphine	–	–	–	–
Diazepam + midazolam + morphine	4 (8.7)	0.4 (0.2–0.45)	0.1 (0.01–0.2)	0.12 (0.08–0.95)
Total	31 (67.39)	0.475 (0.08–1.6)	0.07 (0.01–0.4)	0.155 (0.01–0.95)

**Table 3:** Univariate 0.75 quantile regression model for the % of time of intraoperative hyperoxia_FTOE_ including the demographic and clinical data and the cumulative doses of preoperative sedative agents as potential predictors.

Variable	Regression Beta Coefficient (β)	95% Confidence Interval
Preoperative variables		
Age (days)	−0.45	−1.54; 0.64
Sex (female vs. male)	−6.2	−50.25; 37.85
Weight (g)	−0.01	−0.04; 0.02
Gestation (weeks)	0.63	−10.60; 11.87
Gestation (preterm vs. term)	−3.30	−59.30; 52.70
ASA class (1 vs. 2 vs.3 vs.4)	2.10	−27.54; 31.74
Type of surgery (abdominal vs. other)	−21.4	−60.62; 17.82
Preoperative hemoglobin (g·L^−1^)	0.28	−0.44; 1.00
Preoperative mechanical ventilation (no vs. yes)	−33.4	−103.75; 36.95
24 h preoperative cumulative dose of sedative agents (mg·kg^−1^)	54.96	14.30; 95.62 ^1^
Intraoperative variables		
Duration of surgery (min)	−0.15	−0.64; 0.35
SpO_2_ (%)	2.85	−6.85; 12.55
Arterial blood pressure (mean, mm·Hg)	1.31	−1.27; 3.89
End tidal (ET) CO_2_ (mm Hg)	1.80	−1.25; 4.85
ET sevoflurane concentration (%)	−6.53	−61.36; 48.31
Fentanyl dose (µg·kg^−1^)	9.76	−10.54; 30.05

^1^*p* < 0.009.

**Table 4:** Comparison of the clinical variables between patients given and not given sedative agents preoperatively.

Variable	Patients, Given Sedative Agents (*n* = 31) Median (Range) or *n (%)*	Patients Not Given Sedative Agents (*n* = 15) Median (Range) or *n (%)*
ASA class		
1	1 (3.23)	1 (6.67)
2	11 (35.48)	10 (66.67)
3	15 (48.39)	2 (13.33)
4	4 (12.9)	2 (13.33)
Male sex	19 (61.29)	8 (53.33)
Weight (g)	3224.61 (786.67) ^1^	3659.2 (1001.88) ^1^
Age (days)	6 (0–32)	40 (1–90) ^2^
Abdominal surgery	20 (64.52)	4 (26.67) ^3^
Preterm infants	9 (29.03)	6 (40)
Patients on preoperative mechanical ventilation	5 (16.3)	1 (6.67)
Preoperative Hb (g·L^−1^)	166.42 (23.75) ^1^	136.27 (41.94) ^1,4^
Preoperative blood lactate concentration (mmol·L^−1^)	2.24 (0.88) ^1^	2.27 (0.53) ^1^
Intraoperative SpO_2_ (%)	96.75 (90.51–99.85)	97.53 (87.85–99.96)
Intraoperative arterial blood pressure (mean, mm Hg)	47.37 (9.65) ^1^	48.1 (7.11) ^1^
Intraoperative ET CO_2_	34.12 (7.57) ^1^	36.92 (6.17) ^1^
Intraoperative ET sevoflurane concentration (%)	1.69 (0.4) ^1^	2.08 (0.34) ^1,5^
Intraoperative fentanyl dose (µg·kg^−1^)	3.2 (1.1–7.3)	2.7 (1.0–6.5)
Intraoperative rSO_2_ (%)	80.61 (1.58) ^1^	78.02 (2.35) ^1^

^1^ mean (SD); ^2^ *p* = 0.005 compared to patients given sedative agents; ^3^ *p* = 0.016 compared to patients given sedative agents; ^4^ *p* = 0.003 compared to patients given sedative agents; ^5^ *p* = 0.002 compared to patients given sedative agents.

**Table 5:** Predictive value of the cumulative dose of preoperatively administered sedative agents for the percentage (%) of time of intraoperative hyperoxia_FTOE_ by multivariate 0.75 and 0.5 (median) quantile regression models adjusted for possible confounding variables.

	Regression Beta Coefficient (β)	95% Confidence Interval	*p* Value
24 h preoperative cumulative dose of sedatives (mg·kg^−1^) ^1^	47.12	7.32; 86.92	0.022
24 h preoperative cumulative dose of sedatives (mg·kg^−1^) ^2^	14.26	−15.97; 44.49	0.34

^1^ 0.75 quantile regression β adjusted for patients’ age, preoperative hemoglobin, type of surgery, intraoperative sevoflurane concentration, intraoperative arterial blood pressure, intraoperative dose of fentanyl, and intraoperative end-tidal CO_2_; ^2^ 0.5 quantile regression β adjusted for patients’ age, preoperative hemoglobin, type of surgery, intraoperative sevoflurane concentration, intraoperative arterial blood pressure, intraoperative dose of fentanyl, and intraoperative end-tidal CO_2_.

## Supplementary Materials

The following are available online at https://www.mdpi.com/2227-9067/7/11/209/s1: Table S1: Multivariate 0.75 quantile regression model showing the association of percentage (%) of time of intraoperative hyperoxiaFTOE with sedative agents and confounding clinical variables. Table S2: Multivariate 0.5 (median) quantile regression model showing the association of percentage (%) of time of intraoperative hyperoxiaFTOE with preoperatively administered sedative agents and possible confounding variables.
