# Supplementary material for: Potential Relationship between Cerebral Fractional Tissue Oxygen Extraction (FTOE) and the Use of Sedative Agents during the Perioperative Period in Neonates and Infants
Source: Children (Basel). 2020 Nov 3;7(11):209. doi: 10.3390/children7110209 (PMC7692108; doi:10.3390/children7110209)
Supplement: Supplementary file 1 [file children-07-00209-s001.pdf]

Table S1. Multivariate 0.75 quantile regression model showing the association of percentage (%) of time of intraoperative $hyperoxia_{FTOE}$ with sedative agents and confounding clinical variables.

Variable	Regression beta coefficient (β)	95% confidence interval
24 h preoperative cumulative dose of sedatives (mg kg^{-1})	47.12	7.32; 86.92 [1]
Age (days)	-0.02	-1.16; 1.12
Preoperative hemoglobin (g·L^{-1})	0.25	-0.51; 1.02
Type of surgery (abdominal vs. other)	-2.25	-34.93; 30.43
Intraoperative end tidal (ET) sevoflurane concentration (%)	22.56	-26.83; 71.95
Intraoperative fentanyl dose (µg kg^{-1})	-3.68	-16.01; 8.64
Intraoperative arterial blood pressure (mean, mm Hg)	1.67	-0.20; 3.55
Intraoperative $ETCO_2$ (mmHg)	0.43	-2.27; 3.14

[1] $p = 0.022$

Table S2. Multivariate 0.5 (median) quantile regression model showing the association of percentage (%) of time of intraoperative $hyperoxia_{FTOE}$ with preoperatively administered sedative agents and confounding clinical variables.

Variable	Beta coefficient (β)	95% confidence interval
24 h preoperative cumulative dose of sedatives (mg kg^{-1})	14.26	-15.97; 44.49
Age (days)	-0.22	-1.09; 0.64
Preoperative hemoglobin (g·L^{-1})	0.16	-0.42; 0.74
Type of surgery (abdominal vs. other)	0.91	-23.90; 25.73
ET sevoflurane concentration (%)	-10.48	-47.99; 27.04
Intraoperative fentanyl dose (µg kg^{-1})	0.26	-9.10; 9.63
Intraoperative arterial blood pressure (mean, mm Hg)	0.53	-0.89; 1.96
Intraoperative $ETCO_2$ (mmHg)	1.62	-0.43; 3.68
